# Rheological and Solubility Properties of Soy Protein Isolate

**DOI:** 10.3390/molecules26103015

**Published:** 2021-05-19

**Authors:** Timothy D. O′Flynn, Sean A. Hogan, David F. M. Daly, James A. O′Mahony, Noel A. McCarthy

**Affiliations:** 1 Teagasc Food Research Centre, Food Chemistry & Technology Department, Moorepark, Fermoy, P61 C996 Cork, Ireland; timothy.oflynn@teagasc.ie (T.D.O.); Sean.A.Hogan@teagasc.ie (S.A.H.); 2 School of Food and Nutritional Sciences, University College Cork, T12 YT20 Cork, Ireland; sa.omahony@ucc.ie; 3 Abbott Nutrition, a Division of Abbott Laboratories, Cootehill, H16 RK50 Cavan, Ireland; david.f.daly@abbott.com

**Keywords:** soy protein, thermal treatment, pH, solubility, rheology

## Abstract

Soy protein isolate (SPI) powders often have poor water solubility, particularly at pH values close to neutral, which is an attribute that is an issue for its incorporation into complex nutritional systems. Therefore, the objective of this study was to improve SPI solubility while maintaining low viscosity. Thus, the intention was to examine the solubility and rheological properties of a commercial SPI powder at pH values of 2.0, 6.9, and 9.0, and determine if heat treatment at acidic or alkaline conditions might positively influence protein solubility, once re-adjusted back to pH 6.9. Adjusting the pH of SPI dispersions from pH 6.9 to 2.0 or 9.0 led to an increase in protein solubility with a concomitant increase in viscosity at 20 °C. Meanwhile, heat treatment at 90 °C significantly improved the solubility at all pH values and resulted in a decrease in viscosity in samples heated at pH 9.0. All SPI dispersions measured under low-amplitude rheological conditions showed elastic-like behaviour (i.e., *G*′ > *G*″), indicating a weak “gel-like” structure at frequencies less than 10 Hz. In summary, the physical properties of SPI can be manipulated through heat treatment under acidic or alkaline conditions when the protein subunits are dissociated, before re-adjusting to pH 6.9.

## 1. Introduction

According to the USDA, 360 million metric tons of soybean was produced globally in 2018, ranking it the highest volume oilseed crop in the world [1]. An excellent source of bioavailable protein, soybeans are composed of ≈40% protein, 18–22% oil, and 35% carbohydrate [2,3,4]. Having good amino acid quality, with the exception of methionine, soy protein is often used as a major protein ingredient source in food product formulation [5]. There are four major soybean storage proteins; 2S, 7S, 11S, and 15S, two of which, 7S (β-conglycinin) and 11S (glycinin), are responsible for up to 35 and 52% of the total protein content, respectively [6,7]. These storage proteins are shown to be consumed rapidly during soybean germination for their nitrogen component [8]. Glycinin proteins are hexamers, with molecular masses in the range 320 to 375 kDa, and they are composed of two trimers, containing a number of polypeptide chains linked via disulphide bonds [9]. The β-conglycinin proteins are composed of three homologous proteins with a molecular weight of 76, 72, and 53 kDa and are held together via non-covalent interactions [9].

Soy protein concentrates (SPC) and isolates (SPI) are typically produced through a combination of dry (dehulling and milling) and wet (aqueous alcohol washing and acid precipitation) processing techniques [10]. SPIs generally have a protein content greater than 90%, *w/w*, and are used extensively in nutritional formulations due to their high protein content, neutral taste, and excellent emulsification properties [8,11,12,13]. However, one of the main limiting factors with the utilisation of soy protein is its inherent low solubility [14]. The solubility of SPI is influenced by multiple factors, such as dissolution temperature, pH, and ionic strength [15], with lowest solubility (≈10%) at pH 4.6 (isoelectric point) [16]. Lokuruka (2011) stated that thermal treatment and solvent-based approaches used for protein extraction may influence solubility by denaturing proteins and exposing hydrophobic amino acid residues [17]. Although the solubility of soy protein may not be as high as some animal proteins such as whey, the solubility profile of soy protein allows it to be a good protein ingredient source [18]. The influence of pH on solubility of soy protein has been shown to display a U-shaped profile in which the highest solubility of SPI is achieved on either side of the isoelectric point (pI ≈ 4.6), with complete protein solubility potentially achieved at pH 11 [19]. While Nakai et al. (1980) showed that through the addition of sodium dodecyl sulphate (SDS) and alterations of pH (10% SDS at pH 12, 22 °C for 5 min), the solubility of SPI can be increased by up to 24% [20]. The interaction between hydrophobic groups within the SDS molecule and the hydrophobic amino acid residues on the soy proteins result in an increase in the negative charge of the protein, preventing aggregation and subsequent precipitation. However, simple adjustment of pH to highly acidic or alkaline environments causes a dissociation of the complex protein structure of β-conglycinin and glycinin into their representative subunits, resulting in a significant increase in protein solubility [21,22]. It has also been shown, for a variety of different commercial SPI ingredients, that greater solubility is achieved in heated systems containing higher levels of low molecular weight subunits (10–400 KDa) compared to those containing higher molecular weight (>400 KDa) material [23]. Lee et al. (2003) showed that the solubility of SPI in 0.1 M NaCl at 25 °C did not vary significantly over a range of thermal heat treatment temperatures (i.e., ranging from 25 to 75 °C) [15]. A reason for this may be related to the fact that the temperature range used did not exceed the denaturation temperature of both soy protein subunits.

Aside from challenges associated with solubility, SPI dispersions can exhibit particularly high viscosity. The high viscosity of protein dispersions can often be a limiting factor during processing, as food systems with high viscosity are often responsible for fouling during heat treatment and particularly during water removal and concentration [24,25]. In addition, high viscosity protein dispersions can result in protein material remaining suspended in solution rather than becoming solubilised. This can result in inaccurate soy protein solubility values, which is the reason why a particular focus on soy protein solubility was undertaken in this study. In fact, Liu et al. (2011) showed that SPI dispersions at concentrations of 5%, *w/w*, protein displayed a weak-gel-like property during low oscillatory measurements [26]. These physicochemical properties make soy protein an ideal ingredient for some product applications such as plant-based yogurts, high protein powders, or soy-based beverages in the form of an oil-in-water emulsion; however, to the authors’ knowledge a commercially available soluble soy protein based beverage without oil addition does not exist.

Therefore, based on the previous studies mentioned above, it is expected that the solubility may be improved and viscosity controlled through heat treating SPI dispersions above their denaturation temperatures under acidic or alkaline conditions prior to re-neutralisation, ultimately with the aim of creating a stable dispersion at neutral pH.

## 2. Results and Discussion

### 2.1. Influence of pH on ζ-Potential and Colour of Soy Protein Dispersions

**Figure 1:**
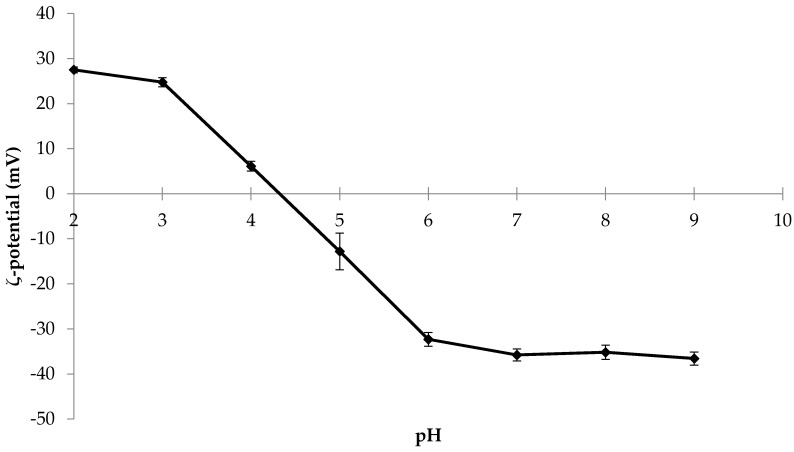
Zeta potential as a function of pH for soy protein isolate dispersions.

The ζ-potential values of dilute SPI dispersions measured as a function of pH ranged from 27.5 mV at pH 2.0 to −36.6 mV at pH 9.0 (Figure 1). At pH values in the range 7.0 to 9.0, the ζ-potential did not change, while a significant decrease in the net negative charge was evident at pH values < 6.0. A cross-over at the isoelectric point occurred at ≈pH 4.2 (i.e., 0 mV) while at pH 4.0, the ζ-potential was +6.0 mV. However, it was not possible to measure ζ-potential at pH values close to the isoelectric point, due to protein precipitation. Then, the net positive charge of SPI dispersions continued to significantly increase up to 24.8 mV at pH 3 and only increased slightly further at pH 2.0 (27.5 mV). Malhotra and Coupland (2004) showed a similar trend for ζ-potential of SPI dispersions as a function of pH, with the ζ-potential reaching 0 mV between pH 4 and 5 [14] and with approximate values of +20 and −50 mV at pH 2 and 8, respectively. Probably the most important point from Figure 1 is that there was no significant increase in the net negative charge from ≈pH 7.0 to 9.0, while it is well understood [22] that an increase in pH results in substantial improvements in solubility, which is often attributed to an increase in electrostatic repulsion.

The effect of pH on the colour profile of SPI dispersions is shown in Table 1. Increasing the pH from pH 6.9 (control pH) to pH 9.0 resulted in decreases in the values for L* (63.8 to 43.5), a* (−2.13 to −3.19) and b* (7.12 to 1.63) parameters, which is indicative of a decrease in whiteness and an increase in yellow and blue hue. The changes in the L^*^ value were partly reversible, as shown in Table 1, once the pH was adjusted back to pH 6.9; however, a* and b* values decreased further. This seems to be in general agreement with the study of Jiang et al. (2010), who found that adjusting the pH of SPI to pH 12, equilibrating for 1 h, before re-adjusting back to pH 7.0 resulted in a lower turbidity than SPI dispersions prepared at pH 7.0 [21]. However, decreasing the pH from 6.9 to 2.0 did not result in a significant change in L^*^ (63.8 to 62.6) or a^*^ values (2.13 to −2.62) with only a minor decrease in the b^*^ values (7.12 to 5.57). This is indicative of the SPI dispersion changing to a slightly darker, green and blue dispersion. After readjustment of pH back to pH 6.9, the L* value reduced further to 57.91, with a^*^ and b^*^ values returning to their original values. Again, this seems to align with data shown by Jiang et al. (2010) when adjusting the pH of SPI to pH 1.5 before re-neutralising to pH 7, resulting in very little change in turbidity between it and SPI dispersions at pH 7.0, when measured at 20 °C [21]. It is well understood that adjusting the pH of SPI dispersions to extremely acidic or alkaline environments causes a significant change in the tertiary and quaternary structure of both β-conglycinin and glycinin, resulting in protein dissociation and the formation of subunits, which will ultimately affect colour properties.

**Figure 2:**
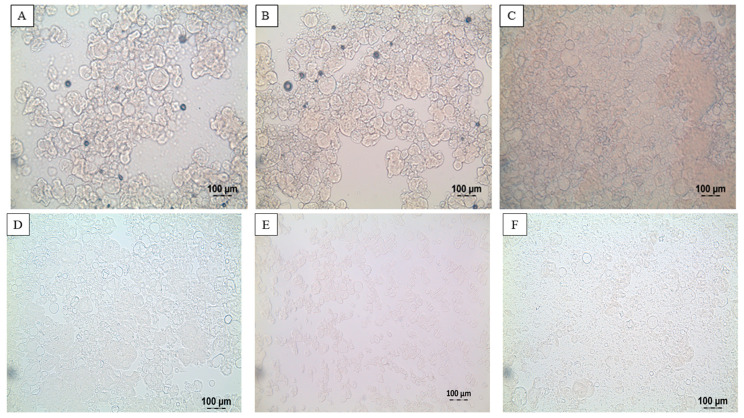
Light microscopy images of soy protein isolate dispersions before heat treatment at pH 2.0 (**A**), pH 6.9 (**B**), and pH 9.0 (**C**) and after heat treatment at 90 °C for 20 min at pH 2.0 (**D**), pH 6.9 (**E**), and pH 9.0 (**F**).

Table 1 also shows the effect of pH on the colour profile of heat-treated SPI dispersions. Comparing the colour of dispersions heat-treated at pH 6.9 to the dispersion at pH 9.0 showed a decrease in the L* (63.8 to 61.19) and a* values (−2.89 to −3.09) with the b* value remaining unchanged (5.46 to 5.41). This was visually represented as a slightly darker dispersion; however, the changes were not as large as their unheated counterparts. Meanwhile, the SPI dispersion heat-treated at pH 2.0 resulted in a decrease of the L* (63.8 to 59.6) and b* values (5.46 to 2.55), and an increase in the a* value (−2.89 to −2.37) compared to the heated dispersion at pH 6.9. This resulted in a darker dispersion with an increase in the blueness corresponding to the decrease in the b* value. After readjustment of the SPI dispersions to the original pH 6.9, the post pH 2 dispersions showed a darker, greener, and yellower appearance, as shown by the decrease in the L* value (63.8 to 60.6) and an increase both a* (−2.89 to −0.97) and b* (5.46 to 8.58) values. The heat-treated pH 9.0 dispersion showed a lighter, redder, and yellower appearance, which was represented as an increase in L* (63.8 to 67.38), a* (−2.89 to −2.03), and b* values (5.46 to 7.27). Light microscopy images (Figure 2) of SPI dispersions measured at pH 2.0, 6.9, and 9.0 before and after heat treatment at 90 °C × 20 min show that heat-treated samples have a less defined particle boundary and appear to be more homogenously distributed throughout the solution. However, particulate material is still clearly visible in all heat-treated dispersions (Figure 2D–F).

### 2.2. Particle Size and Protein Solubility Analysis of Soy Protein Dispersions

**Figure 3:**
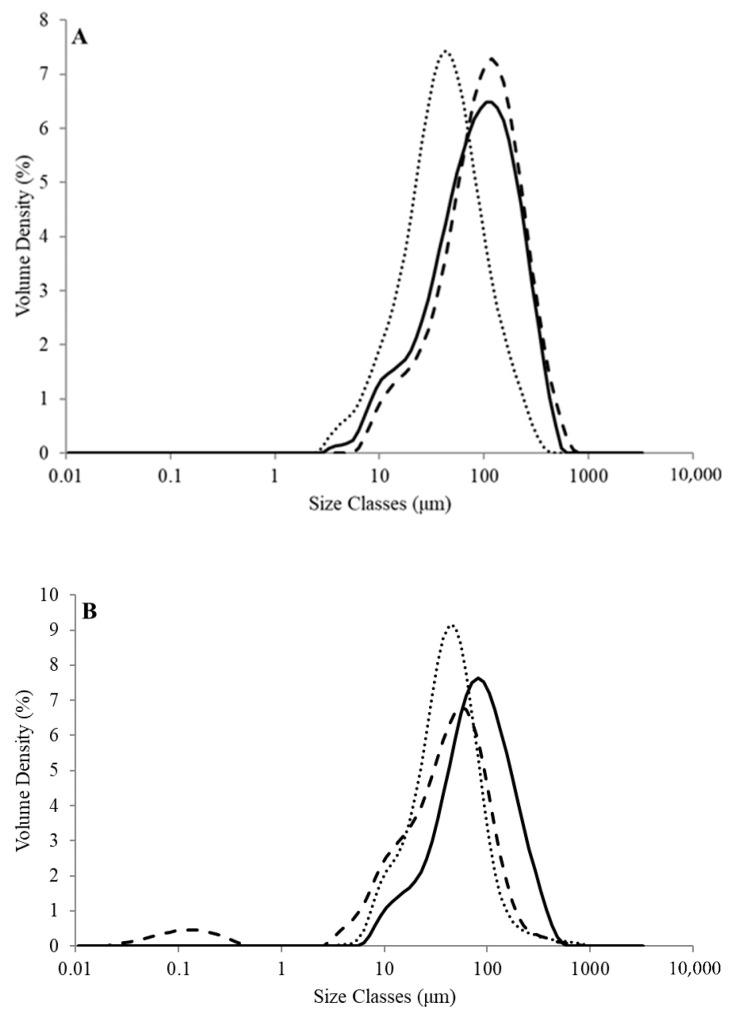
Particle size of soy protein isolate dispersions without (**A**) and with heat treatment (**B**) (90 °C × 20 min) at pH 6.9 (**――**), pH 9.0 (─ ─ ─) and pH 2.0 (‧‧‧‧).

Particle size distribution profiles of unheated and heated (90 °C × 20 min) SPI dispersions are shown in Figure 3. The size of the particles in unheated SPI dispersions ranged from 10 to 600 μm, with all dispersions displaying monomodal distributions (Figure 3A), similar to a number of previous studies [27,28]. Unheated and heated SPI dispersions at pH 6.9 had weighted mean values (*D*_4,3_) of 130 and 101 μm, respectively, with unheated and heated SPI dispersions at pH 2.0 having significantly (*p* < 0.05) smaller *D*_4,3_ values of 57.2 and 52.1 μm, respectively. The mean particle size was significantly (*p* < 0.05) smaller in the heated SPI dispersions at pH 9.0 (*D*_4,3_ = 54.6 μm) than the respective unheated samples (*D*_4,3_ = 128 μm), with a bimodal size distribution (Figure 3B). The particle size data presented in the current study are comparable to those of other plant protein dispersions, such as those from pea and fava bean protein [29,30]. The reduction in particle size in heated dispersions at pH 2.0 and 9.0 indicate that some protein dissociation did occur but certainly not complete break-down into individual subunits. Peng et al. (2020) found a similar particle size distribution profile for unheated soy protein dispersions measured across a range of pH values [22].

**Figure 4:**
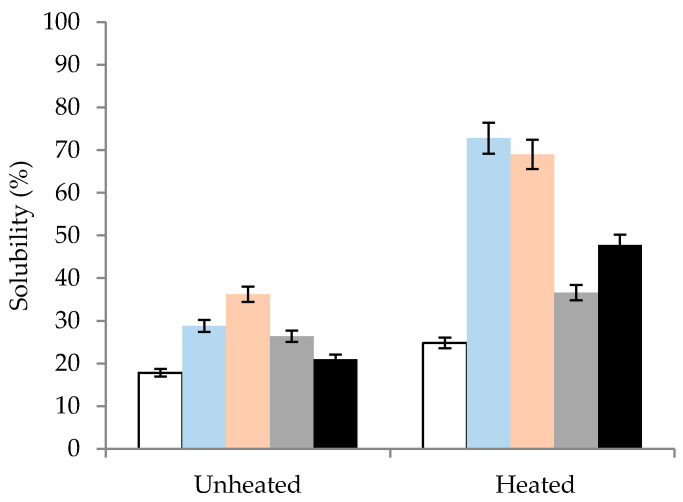
Solubility of unheated and heated (90 °C × 20 min) soy protein isolate dispersions measured at pH 6.9 (□), pH 9.0 (■), pH 2.0 (■), and pH 6.9 readjusted after heat treatment at pH 9.0 (■) and pH 6.9 readjusted after heat treatment at pH 2.0 (■).

To further investigate the effect of heating on the functional properties of SPI dispersions and extrapolate the reduction in particle size to solubility, dispersions were centrifuged at 800 *g* and the solids content suspended in the supernatant was measured. The solubility of SPI dispersions was significantly influenced by changes in both pH and thermal treatment (Figure 4). The solubility of unheated SPI dispersions at pH 9.0 (28.8%) was significantly higher than in dispersions at pH 6.9 (17.8%). However, when the pH was reduced from 9.0 to 6.9, a slight decrease in solubility was observed (i.e., 26.4%; Figure 4). The solubility of unheated SPI dispersions at pH 2.0 was 36.2%, which was higher than the 21% solubility when the pH was readjusted back to pH 6.9. It has previously been reported [30,31] that the solubility of SPI can be displayed as a U-shaped curve at which the lowest point is close to the isoelectric point between pH 4.0 and 5.0, with near 100% solubility achieved at both pH 2.0 and pH 9.0. However, the solubility values shown in the present study at pH 2.0 and 9.0 are lower than the values presented in the aforementioned studies. This may be due to the differences in the SPI production processes, whereby in the present study, a commercially produced SPI was used, as opposed to native soy protein.

Heat treatment was shown to increase the solubility of all samples compared to their respective unheated samples (Figure 4), with solubility values of 24.8, 72.8, and 69.0% for heated dispersions at pH 6.9, 9.0, and 2.0. This may be contrary to some studies where the thermally induced denaturation/aggregation may be associated with a reduction in solubility; however, as far back as Kinsella (1979) [32], who stated that while it is commonly acknowledged that low heat treatment equates to better solubility, it may not always be the case. This in part is due to changes in the protein structure, particularly the glycinin fraction, at temperatures greater than the denaturation temperature. However, what seems to be more significant is the pH at which heat treatment is performed. Heat-treating soy protein in a semi-dissociated state at extremes of pH alters the structure of the protein, allowing the solvent access to the newly exposed pockets of secondary structures within the protein, aiding hydration and solubilisation. These results are also in agreement with Jiang et al. (2010), who reported that the dissociation of the SPI complexes at 21 °C can lead to the exposure of hydrophobic groups previously occluded in the native agglomerates [21]. They also reported a restructuring of protein secondary structural characteristics in the 11S and the 7S subunits after exposure to extreme pH conditions (pH 1.5 or pH 12) for 1 h at room temperature before pH readjustment back to pH 7. Interestingly, the ζ-potential remained relatively constant when changing the pH from 6.9 to 9.0, as shown in Figure 1, indicating that electrostatic repulsion between amino acid subunits is obviously not the only contributory factor for the increased solubility under severe alkaline conditions. Readjusting the pH from 9.0 and 2.0 to 6.9 after heat treatment resulted in solubility values of 36.6 and 47.8%, respectively, which is a decrease in solubility compared to the solubility at pH 2.0 and 9.0, but it may be attributed to the partial re-folding of the protein subunits. Another point to mention is that the addition of NaOH and HCl during pH adjustment may increase the ionic strength or NaCl content of the dispersions. However, the amounts added would seem to contribute very little effect on the actual SPI solubility, as Jiang et al. (2010) [21] found that only NaCl concentrations ≥100 mM, substantially higher than in the present study, had a significant effect on SPI solubility, which was attributed to a decrease in electrostatic repulsion.

### 2.3. Influence of pH and Heat Treatment on Viscosity of Soy Protein Dispersions

**Figure 5:**
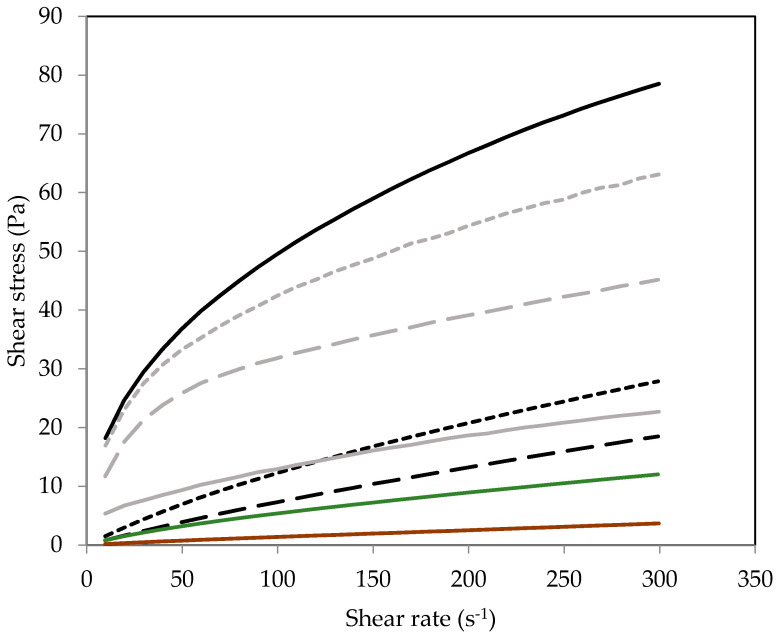
Flow curves of unheated (Black) and heat-treated (90 °C × 20 min) (Grey) soy protein isolate dispersions (10%, *w/w*) at pH 2.0 (**- - - -**; **- - - -**), pH 6.9 (**–– –– ––**;**–– –– ––**) and pH 9.0 (**―**; **―**), and soy protein isolate dispersions measured at pH 6.9 after heat treatment at pH 2 (**—**) and pH 9 (**—**).

**Figure A1:**
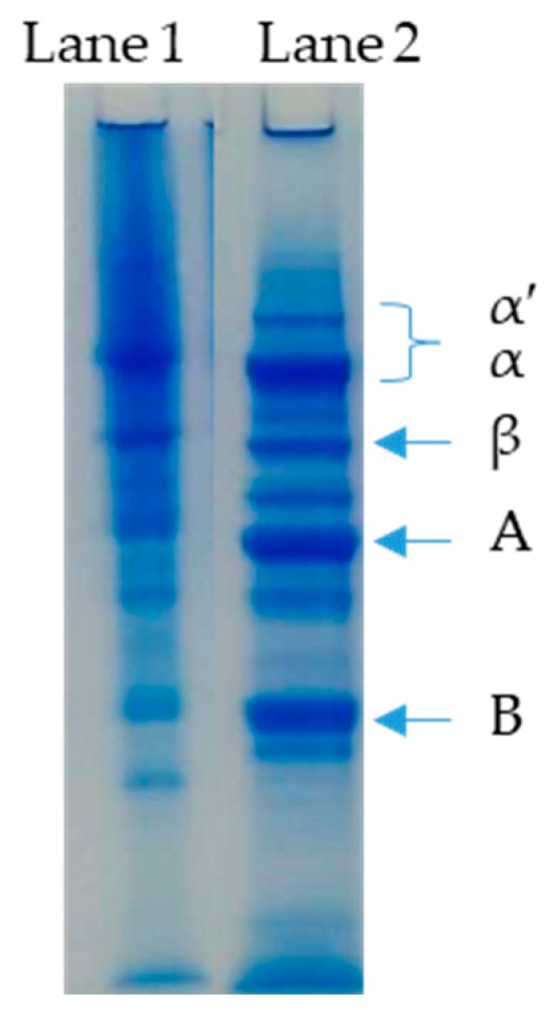
Sodium dodecylsulphate polyacrylamide gel electrophoresis (SDS-PAGE) profile of soy protein isolate obtained under non-reducing (Lane 1) and reducing (Lane 2) conditions.

Viscosity profiles for SPI dispersions at pH 2.0, 6.9, and 9.0 before and after heat treatment (90 °C × 20 min) are shown in Figure 5. The heat treatment of 90 °C for 20 min was selected as the denaturation temperature for both major components of SPI occurs below or at ≈90 °C [22,33]. Note that while the exact production conditions of the commercial SPI (heat treatment, pH-induced precipitation, etc.) are unknown, the thermal treatment applied in this study was deemed sufficient to exceed denaturation/aggregation conditions. A sodium dodecylsulphate polyacrylamide gel electrophoresis (SDS-PAGE) profile of the SPI indicates some initial protein denaturation, as shown by a decrease in protein band intensity obtained under non-reducing conditions (Appendix A.2: Figure A1; lane 1) compared to those observed in the reducing lane (Appendix A.2: Figure A1; lane 2). For all unheated and heated SPI dispersions, the shear stress increased with increasing shear rate (Figure 5), indicating that the dispersions were non-Newtonian shear-thinning fluids (Figure 5). Table 2 data show that the flow behaviour index (*n*) was initially 0.86 for unheated SPI dispersions at pH 6.9 and was lower for SPI dispersions at pH 2 (*n* = 0.76) and pH 9 (*n* = 0.43). However, after heat treatment (90 °C × 20 min), the *n* values decreased in SPI dispersions at pH 6 and pH 2.0, but they remained constant at pH 9.0. This decrease in *n* value indicates an increase in shear-thinning behaviour. The consistency coefficient (*K*) values of SPI dispersions at pH 6.9 and pH 2.0 were 0.14 and 0.36 Pa.s, respectively, whereas at pH 9.0, the *K* value was significantly higher at 6.99 Pa.s, indicating a higher overall viscosity. Conversely, for SPI dispersions measured after the heat treatment, an inverse in *K* values was observed, with samples at pH 6.9, 2.0, and 9.0 having *K* values of 6.97, 7.90, and 1.42 Pa.s, respectively.

At pH 6.9, the shear stress of unheated SPI dispersions increased from 0.8 to 18.5 Pa over a shear rate ramp of 9.6 to 300 s^−1^, while adjusting the pH from 6.9 to 2.0 resulted in an increase in shear stress from 1.5 to 27.9 Pa (Figure 5). The most significant difference (*p* < 0.05) amongst the unheated samples was the SPI dispersion adjusted to pH 9.0, which showed a significantly higher viscosity than dispersions at pH 2.0 and pH 6.9 (Figure 5). Initially, the shear stress was 18.2 Pa at a shear rate of 9.6 s^−1^ and increased up to 78.5 Pa at 300 s^−1^. This may be due to the dissociation of β-conglycinin (7S) and glycinin (11S) into subunits and an increase in protein polarity at the high pH, resulting in a concomitant increase in water-binding capacity. Previously, a study showed an increase in water-holding capacity in commercial soy protein from ≈2 up to 10 g of water/g of protein when increasing the pH from 3.5 to 7.5 [22]. Heat-treated SPI dispersions at pH 6.9 and pH 2.0 had a significantly higher viscosity compared to unheated samples, which was attributed to the additional denaturation/aggregation of the two major protein fractions in SPI, β-conglycinin and glycinin, having denaturation temperatures of ≈72 and 90 °C, respectively [6]. However, heating SPI at pH 9.0 was the only sample to result in a lower viscosity compared to its unheated counterpart (Figure 5 and Table 2). Native glycinin is a hexamer [34] comprised of six subunits with each subunit made up of an acidic and basic peptide (covalently linked via a disulphide bond); as a result, it is the non-covalent bonds (hydrophobic and electrostatic interactions) between the subunits, which account for the differences in solubility and viscosity across the pH scale [35,36]. Under extreme acidic or alkaline conditions, the non-covalent linkages weaken, and dissociation of the subunits can occur. This dissociation can result in the exposure of previously buried hydrophobic residues, and in conjunction with high negative electrostatic repulsion, it can cause high viscosity in SPI dispersions. However, after heating to 90 °C at pH 9.0, the exposed hydrophobic residues may result in the re-arrangement of the subunits into a more soluble aggregated form, causing a significant decrease in viscosity. Similarly, β-conglycinin comprised of three subunits, α, α′, and β, linked via non-covalent interactions can dissociate as the electrostatic repulsion between them increases at pH values away from the isoelectric point.

### 2.4. Temperature Related Viscoelastic Properties of Soy Protein Dispersions

**Figure 6:**
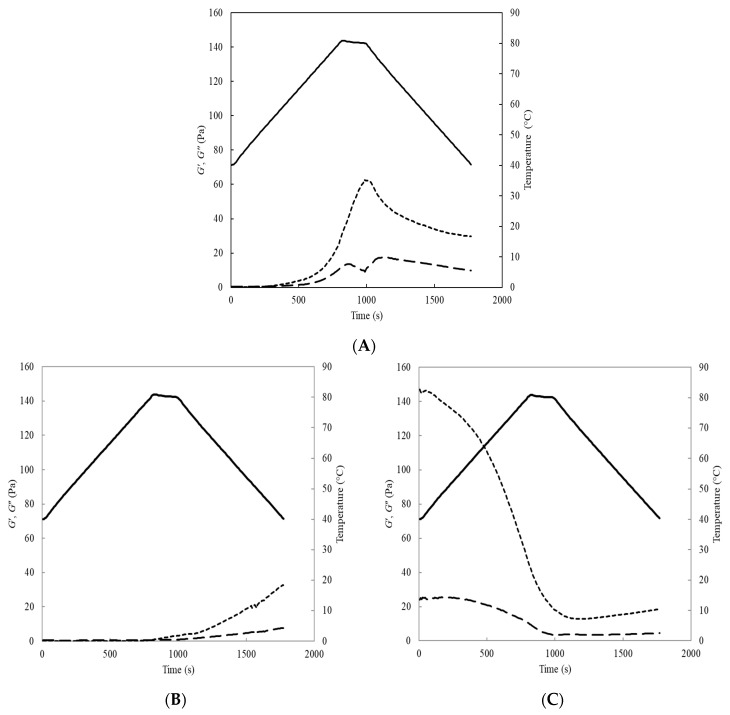
Elastic (*G*′; **---**) and viscous (*G*″; **─ ─**) modulus of soy protein isolate dispersions (10%, *w/w*) measured at pH 2.0 (**A**), pH 6.9 (**B**) and pH 9.0 (**C**), as a function of temperature (**―**).

To investigate the heating and cooling effect on the visco-elastic properties of SPI dispersions, low-amplitude oscillatory measurements were carried out as a function of temperature (Figure 6). SPI dispersions at pH 2.0 showed more elastic than viscous properties as the temperature increased above 65 °C. The *G*’ increased rapidly reaching a maximum of 62.7 Pa at 80 °C. Thereafter, the *G*′ decreased sharply to 29.7 Pa as the temperature was reduced from 80 to 40 °C. A similar trend was measured for the *G*″, as temperature increased, so too did the viscous moduli, peaking at 13.6 Pa, before reducing when the temperature peaked at 80 °C. As temperature started to decrease, *G*″ increased before peaking at 17.4 Pa at 73 °C. The data are indicative of the formation of a weak gel within the SPI dispersion (Note: all SPI dispersions displayed flow properties under high shear after heat treatment). The results of the current study are in broad agreement with those of previous studies [29,37], in which weak gel-like structures were observed in pea and soy protein emulsions, respectively. SPI dispersions at pH 6.9 showed contrasting behaviour, in which *G*′ was initially lower than *G*″ until the temperature peaked at 80 °C, after which *G*′ increased sharply, peaking at 32.8 Pa at 40 °C. *G*″ followed a similar trend, and it remained lower than the *G*′, increasing to only 7.64 Pa. Conversely, the SPI dispersion at pH 9.0 showed a profile in which the *G*′ was initially higher than the *G*″ value, at 147 and 23.9 Pa, respectively. This may be due to the dissociation of soy protein into subunits, causing an increase in the critical protein volume fraction. However, both *G*′ and *G*″ moduli decreased as temperature increased, with *G*′ decreasing to 12.7 Pa at 70 °C, before increasing to 18.6 Pa upon cooling to 40 °C. SPI dispersions at pH 9.0 displayed rheological behaviour consistent with a weak gel as *G*′ remained greater than *G*″ throughout the temperature ramp. An explanation for the decrease in viscosity of the pH 9 dispersion may be due to the substantial changes in protein structure and exposure of hydrophobic groups after extreme alkaline treatment [38].

**Figure 7:**
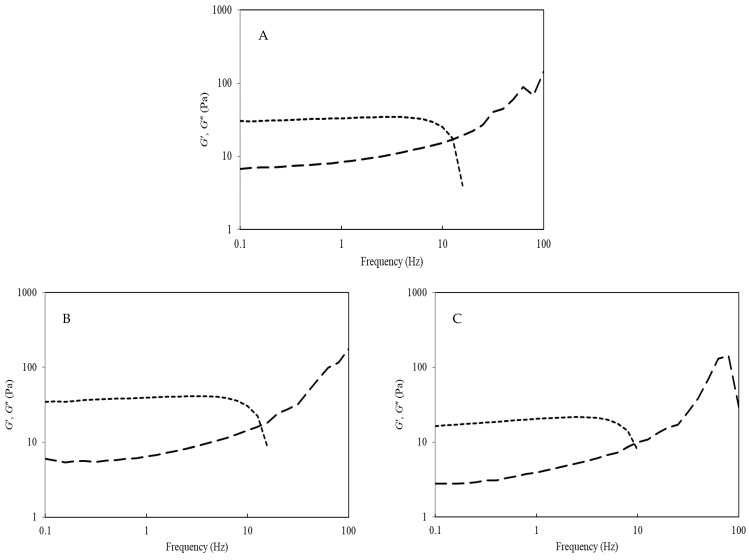
Elastic (*G*′; ---) and viscous (*G*″; **─ ─**) moduli of soy protein isolate dispersions at pH 2.0 (**A**), pH 6.9 (**B**), and pH 9.0 (**C**), measured as a function of frequency (Hz) at 40 °C.

Frequency sweep profiles of SPI dispersions at pH 2.0, 6.9, and 9.0 are shown in Figure 7. All SPI dispersions initially showed *G*′ greater than *G*″, until approximately 10 Hz, indicating that up until this point, a weak gel network existed. Thereafter, the *G*″ > *G*′ for all dispersions, signifying the transition from a weak gel to a more viscous liquid-like system [39]. Previously, Liu et al. (2011) showed that the critical concentration of an SPI dispersion to form a weak gel-like structure was approximately 5% (*w/w*, protein), with protein concentrations less than 5% (*w/w*) showing only viscous behaviour (i.e., *G*″ > *G*′) [26]. Ultimately, the data in Figure 7 confirm that the SPI dispersions have elastic-like behaviour at low frequencies but convert to viscous type systems at frequencies of ≈10 Hz or higher. This may have important knock-on effects in terms of beverage stability, particularly around hindering protein sedimentation during storage. Previously, Malhotra and Coupland (2004) showed good correlation between increased solubility with increasing viscosity in 5%, *w/w*, SPI dispersions [14].

## 3. Materials and Methods

### 3.1. Materials

Non-hydrolysed soy protein isolate (SPI) powder was obtained from a commercial supplier. The total protein content, measured using Kjeldahl analysis (IDF 20-3:2004) of the SPI powder was 88.0%, *w/w*, using a conversion factor of 6.25. Non-protein nitrogen content, measured according to the IDF standard method (Kjeldahl analysis 20-4:2001) was 0.26%, *w/w*. Moisture and fat content was 4.39 and 4.51%, *w/w*, respectively, and it contained calcium (0.18%, *w/w*), sodium (1.1%, *w/w*), magnesium (0.04%, *w/w*), potassium (0.15%, *w/w*), chloride (0.29%, *w/w*), and phosphorous (0.75%, *w/w*), as obtained from the commercial supplier. The pH of rehydrated SPI (5%, *w/w*) was pH 6.9 and was considered as the control pH for all other analysis. An SDS-PAGE protein profile of the SPI is described and shown in Appendix A.

### 3.2. Zeta Potential of Soy Protein Dispersions

Soy protein dispersions were prepared by adding SPI powder to deionised (DI) water at 5%, *w/w*, total solids (20 °C). The suspension was agitated using a magnetic stirrer (Dynalon Stir Bar Kit, VWR, Blanchardstown, Dublin, Ireland) at 350 rpm for 1 h. After 1 h of stirring, these dispersions were centrifuged at 800× *g* for 10 min, and the supernatant was removed. The initial pH of the dispersion was pH 6.9. Then, the pH was adjusted using HCl or NaOH, ranging from pH 2.0 to 9.0. Then, the solids content was diluted to a final concentration of 0.002%, *w/w*, using DI water and an Eppendorf 5417R centrifuge (Eppendorf AG, 22331 Hamburg, Germany). Samples were measured using a Zetasizer Nano ZS (Malvern Instruments Ltd., Worcestershire, UK). All measurements were carried out at 25 °C, in triplicate, in 10 mm polystyrene cuvettes using a refractive index of 1.45.

### 3.3. Light Microscopy of Soy Protein Dispersions

Soy protein dispersions were prepared by adding SPI powder to deionised (DI) water at 5%, *w/w*, total solids (20 °C) as described in Section 3.2 above but without the centrifugation step. The pH of SPI dispersions was adjusted from pH 6.9 using ≈3.5 mL of 1 M HCl or 1 M NaOH to pH 2.0 and 9.0, respectively. Then, sub-aliquots of samples were heat treated at 90 °C for 20 min in 50 mL Falcon tubes (Merck, Wicklow, Ireland) using a controlled-temperature water bath (GFL 1003, 30927 Burgwedel, Germany). The microstructure of unheated and heated samples was examined using an Olympus BX51 light microscope (Olympus BX-51, Olympus Corporation, Tokyo, Japan) under a 10× dry objective lens using differential interference contrast (DIC) at 20 °C. Images were taken using a Jenoptik C14 Imagic camera, and captured and analysed using Image Access Premium^®^ 7 software (Wiltshire, UK).

### 3.4. Colour Analysis of Soy Protein Dispersions

The colour of each dispersion (i.e., as prepared in Section 3.3) was expressed as L*, a*, and b* values using a Minolta Chroma Meter CR-400 colorimeter (Minolta Ltd., Milton Keynes, UK). SPI dispersions were prepared (5%, *w/w*, total solids), and their respective pH values were adjusted using NaOH and HCl accordingly. The dispersions were mixed at 20 °C for 15 min before measurements were performed. The L* value indicates lightness, a* values indicate redness–greenness, and b* values indicate yellowness–blueness. Analysis was performed in triplicate.

### 3.5. Particle Size and Solubility of Soy Protein Dispersions

Soy protein dispersions (5%, *w/w*, total solids) were prepared as described in Section 3.3 above. The solubility analysis of SPI dispersions was performed by placing aliquots in 50 mL centrifuge tubes and centrifuging in a Thermo Scientific™, Heraeus^TM^ Multifuge X1R Centrifuge (Thermo Scientific™, Waltham, MA, USA) at 800 *g* × 5 min at 25 °C. Solubility was expressed as the total solids content of the supernatant expressed as a percentage of the total solids content of the samples prior to centrifugation using a moisture analyser (CEM Smart System5™, Matthews, NC, USA). Measurements were performed in triplicate.

Particle size distribution analysis of SPI dispersions was performed in triplicate using a laser light diffraction unit (Mastersizer, Malvern Instruments Ltd., Worcestershire, UK), equipped with a 300 RF lens. Particle and dispersant (i.e., water) refractive indices were set at 1.48 and 1.33, respectively. Measurements were recorded at laser obscuration levels >4% and at ~20 °C.

### 3.6. Viscosity and Low-Amplitude Oscillatory Measurements of Soy Protein Dispersions

Soy protein dispersions (10%, *w/w*, total solids) were prepared by adding SPI powder to DI water (20 °C). The pH of SPI dispersions were adjusted from pH 6.9 using 1 M HCl or 1 M NaOH to pH 2.0 and 9.0, respectively. Then, sub-aliquots of samples contained in 50 mL conical Falcon tubes (Fisher Scientific, Bishop Meadow Road, Loughborough, Leicestershire, UK) were heat treated at 90 °C for 20 min by placing samples in a temperature-controlled water bath (GFL 1003, 30927 Burgwedel, Germany). Viscosity measurements of dispersions were performed at 25 °C using a controlled stress rheometer (TA Instruments, Crawley, UK), equipped with a concentric cylinder geometry performed at a shear rate ranging from 9.6 to 300 s^−1^ over 3 min. Viscosity measurements were performed on samples before and after heat treatment.

The power law applied to the log–log plots of shear stress (*τ*) versus shear rate (*R*) was used to analyse non-Newtonian fluids as described by:

*τ* = *KR^n^*

where *τ* is the shear stress (Pa), *R* is the shear rate (s^−1^), *K* is the consistency coefficient (Pa.s^n^), and *n* is the flow behaviour index [34]. Values of *n* = 1, *n* < 1, and *n* > 1 indicate Newtonian, shear thinning, and shear thickening flow behaviour, respectively [35].

Low-amplitude oscillatory measurements of unheated SPI dispersions (10%, *w/w*) were analysed using the same rheometer and geometry as above at pH values of 2.0, 6.9, and 9.0. The temperature was increased from 40 to 80 °C at a gradient of 2.5 °C min^−1^, held at 80 °C × 3 min, and decreased to 40 °C at 2.5 °C min^−1^. Oscillatory measurements were recorded at a frequency of 1 Hz and 0.5% strain, giving both the storage (*G*′) and loss (*G*′′) moduli. Subsequently, a mechanical spectrum was performed at a frequency ranging from 0.1 to 100 Hz (frequency dependence of elastic, *G*′, and viscous, *G*″, moduli) at 25 °C (0.5% strain). Frequency sweeps were determined in triplicate within the linear viscoelastic region to ensure non-destructive rheological analysis.

### 3.7. Statistical Analysis

Analysis of variance (ANOVA) was carried out using the Minitab 17 (Minitab Ltd., Coventry, UK, 2007) statistical analysis package, and the effects of treatment and replicates were estimated for response variables. Tukey’s one-way multiple comparison test was used as a guide for pair comparisons of the treatment means. The level of significance was determined at *p* < 0.05.

## 4. Conclusions

This work has demonstrated that the solubility of SPI dispersions can be modified significantly using a combination of pH adjustment and heat treatment. Adjusting the pH of SPI dispersions to acidic (pH 2.0) or alkaline (pH 9.0) conditions increased solubility, with heat treatment at these pH values working synergistically to increase solubility even further. The use of acidic/basic pH conditions influences the hydroelectric charge on the soy proteins causing subunit dissociation, and in doing so facilitates alternative protein–protein interactions which would otherwise not be possible. This study has shown that the relatively simple adjustment of pH prior to thermal heat treatment followed by pH readjustment can result in a more soluble protein dispersion at neutral pH values, with pH 2 treated dispersions showing a greater solubility after heat treatment and re-neutralisation. Knowledge of these underlying properties, which are responsible for SPI dispersion behaviour, allow for the manipulation of protein functionality in beverage products (i.e., reduced viscosity and greater solubility), and should provide a platform for further work in this area. Particularly, as SPI is often subjected to multiple heating steps during the production of nutritional formulations, the optimal stage in the process for heat treatment at acidic/alkali conditions may yet need to be elucidated.

## Figures and Tables

**Table 1:** Color chromaticity coordinates of unheated and heated (90 °C × 20 min) soy protein isolate dispersions at pH 2.0, pH 6.9, and pH 9.0 and dispersions readjusted from pH 9.0 and pH 2.0 back to pH 6.9.

	pH 2.0	pH 6.9	pH 9.0	pH 6.9 Adjusted after pH 2.0	pH 6.9 Adjusted after pH 9.0
Unheated					
L*	62.6 ± 0.60 ^a^	63.8 ± 0.67 ^a^	43.5± 0.04 ^c^	57.9 ± 4.26 ^b^	59.2 ± 0.47 ^b^
a*	−2.62 ± 0.07 ^b^	−2.13 ± 0.07 ^b^	−3.19 ± 0.03 ^c^	−1.92 ± 0.19 ^a^	−4.07 ± 0.0 ^d^
b*	5.57 ± 0.07 ^b^	7.12 ± 0.22 ^a^	1.63 ± 0.03 ^c^	7.36 ± 0.19 ^a^	0.08 ± 0.08 ^d^
Heated					
L*	59.6 ± 0.83 ^b^	63.8 ± 2.58 ^a^	61.2 ± 0.58 ^b^	60.6 ± 0.94 ^b^	67.4 ± 0.55 ^a^
a*	−2.37 ± 0.19 ^b^	−2.89 ± 0.29 ^b^	−3.09 ± 0.03 ^c^	−0.97 ± 0.21 ^a^	−2.03 ± 0.12 ^b^
b*	2.55 ± 0.27 ^d^	5.46 ± 0.07 ^c^	5.41 ± 0.03 ^c^	8.58 ± 0.38 ^a^	7.27 ± 0.32 ^b^

Values are the means of data ± standard deviations with values within a row not sharing a common superscript differing significantly (*p* < 0.05). L*-value denotes white-black, a*-value denotes green-red, b*-value denotes yellow-blue.

**Table 2:** Rheological parameters, consistency coefficient, and flow behavior index of soy protein isolate dispersions (10%, *w/w*, protein) as a function of pH before and after heat treatment.

pH	Consistency Coefficient	Flow Behavior Index
	*K* (Pa.s)	*n*
pH 6.9	0.14 ± 0.01 ^e^	0.86 ± 0.02 ^b^
pH 2.0	0.36 ± 0.00 ^d^	0.76 ± 0.03 ^c^
pH 9.0	6.99 ± 0.01 ^b^	0.43 ± 0.02 ^d^
pH 6.9 *	6.97 ± 0.07 ^b^	0.33 ± 0.02 ^e^
pH 2.0 *	7.90 ± 0.04 ^a^	0.36 ± 0.03 ^e^
pH 9.0 *	1.42 ± 0.00 ^c^	0.48 ± 0.02 ^d^
pH 6.9 after heating @ pH 9.0	0.16 ± 0.00 ^e^	0.74 ± 0.01 ^c^
pH 6.9 after heating @ pH 2.0	0.02 ± 0.00 ^f^	0.90 ± 0.03 ^a^

* Samples heated at 90 °C × 20 min. Values are the means of data ± standard deviations; ^a–f^ values within a column not sharing a common superscript differ significantly (*p* < 0.05).

## Appendix A

### Appendix A.1. Sodium Dodecylsulphate Polyacrylamide Gel Electrophoresis

The protein profile of the SPI powder was determined using sodium dodecylsulphate-polyacrylamide gel electrophoresis (SDS-PAGE). The powder sample was dissolved under reducing and non-reducing conditions in an SDS buffer; 10 μL of each buffer was added to wells in a 12% Bis-Tris Nu-PAGE gel and electrophoretic analysis was performed using an X-Cell Surelock electrophoresis unit (Novex Technologies, Fischer Scientific, Blanchardstown, Dublin, Ireland) at a constant voltage of 180 V for 55 min. Samples for SDS-PAGE analysis contained 1 μg protein per μL of sample buffer solution. Gels were then stained overnight using 0.05% (*w/v*) Coomassie brilliant blue R-250 in 25% (*v/v*) isopropanol and 10% (*v/v*) acetic acid. After staining, the gels were de-stained using a 10% (*v/v*) isopropanol and 10% (*v/v*) acetic acid solution until a clear background was achieved.
